# A nutrition strategy to reduce the burden of diet related disease: access to dietician services must complement population health approaches

**DOI:** 10.3389/fphar.2015.00160

**Published:** 2015-08-11

**Authors:** Leonie Segal, Rachelle S. Opie

**Affiliations:** ^1^Health Economics and Social Policy Group, Division of Health Sciences, University of South Australia, Adelaide, SA, Australia; ^2^School of Allied Health, College of Science, Health and Engineering, La Trobe University, Melbourne, VIC, Australia

**Keywords:** nutrition strategy, dietician services, diet quality, population health

## Abstract

Poor diet quality is implicated in almost every disease and health issue. And yet, in most advanced market economies diet quality is poor, with a minority meeting guidelines for healthy eating. Poor diet is thus responsible for substantial disease burden. Societies have at their disposal a range of strategies to influence diet behaviors. These can be classified into: (i) population level socio-educational approaches to enhance diet knowledge; (ii) pricing incentives (subsidies on healthy foods, punitive taxes on unhealthy foods); (iii) regulations to modify the food environment, and (iv) the provision of clinical dietetic services. There is little evidence that societies are active in implementing the available strategies. Advertising of “junk foods” is largely unchecked, contrasting with strict controls on advertising tobacco products, which also attract punitive taxes. Access to dieticians is restricted in most countries, even in the context of universal health care. In Australia in 2011 there were just 2,969 practicing dieticians/nutritionists or 1.3 clinicians per 10,000 persons, compared with 5.8 physiotherapists per 10,000 persons, 14.8 general practitioners (family physicians) per 10,000 persons or 75 nurses per 10,000 persons. It is time to implement comprehensive national nutrition strategies capable of effecting change. Such strategies need to be multi-component, incorporating both public health approaches and expanded publicly funded dietetic services. Access to individualized dietetic services is needed by those at risk, or with current chronic conditions, given the complexity of the diet message, the need for professional support for behavior change and to reflect individual circumstances. The adoption of a comprehensive nutrition strategy offers the promise of substantial improvement in diet quality, better health and wellbeing and lower health care costs.

## Diet Quality and Health

It is uncontentious that diet quality has a major impact on health. This is not simply relational, which it certainly is—populations with better diet quality are shown consistently to have better health outcomes (Keys et al., 1986; Sofi et al., 2008; Lai et al., 2014), but it is undeniably causal. Causality has been demonstrated from rigorous studies investigating causal pathways whereby specific nutrients affect health (Parletta et al., 2013) and through the success of high quality intervention trials such as those incorporating the Mediterranean diet (de Lorgeril et al., 1999; Estruch et al., 2013).

The beneficial effects of a healthy dietary pattern derive from the cumulative and synergistic effect of nutrients from varying food sources. A healthy dietary pattern is widely agreed to be one that is high in fruit and vegetables, whole-grains and fish. Whereas, an unhealthy dietary pattern is high in fried foods and processed meats, refined grains and “extras” such as sugar laden drinks and confectionary (NHMRC, 2014). The key nutrients or foods responsible for the observed health benefits include (but are not limited to) antioxidants, omega-3 polyunsaturated fatty acids (PUFAs) and B vitamins (Parletta et al., 2013). The antioxidants in fruit and vegetables have a protective effect for health and the high content of omega-3 PUFAs from fish have vascular and anti-inflammatory properties (Akbaraly et al., 2009). Some of the health benefits of a recommended diet are now understood to operate through the gut microbiome, which is emerging as an important issue in human health and the development of chronic diseases (Kouris-Blazos and Itsiopoulos, 2014). Unhealthy diet patterns can be associated with pro-inflammatory changes, endothelial dysfunction and insulin resistance (Sánchez-Villegas et al., 2013). A poor quality diet involving excess saturated fat, *trans*-fatty acids, and omega-6 PUFAs has a high glycaemic load and provides insufficient fiber and essential micronutrients (Simopoulos, 2011). A full understanding of how key nutrients affect health is complex, and beyond the scope of this paper.

The effect of diet on health is observed at all stages of disease. It is implicated in the development of disease, as demonstrated in trials of dietary interventions to reduce the incidence of type 2 diabetes in persons with impaired glucose tolerance (Eriksson and Lindgärde, 1991), or in the incidence and prevalence of depression (Opie et al., 2013; Sánchez-Villegas et al., 2013). Diet quality is implicated in the rate of complications, including in those with advanced disease. The seminal Lyon Heart study of persons following a first heart attack (AMI), found that the risk of a major cardiac event was reduced by 66% for those randomized to a Mediterranean diet (de Lorgeril et al., 1999). Diet quality has been demonstrated to affect the rate and quality of recovery after major surgery (Cerantola et al., 2011) and is a critical factor in successful aging (Hodge et al., 2014).

The evidence is clear that diet quality, including diet composition (e.g., macro and micronutrient profile) is critical to health. Diet plays a role in disease incidence, development of complications, disease management, recovery, and quality of life. Diet quality is pertinent across virtually all health conditions; including cancers (Sofi et al., 2008), cardiovascular disease (Estruch et al., 2013), diabetes (Esposito et al., 2010), neurological (including Parkinsons and Alzheimers; Sofi et al., 2008), mental health (Sánchez-Villegas et al., 2011), maternal and child health (Thorton et al., 2009), and gastrointestinal disorders (Heizer et al., 2009).

Obesity (i.e., BMI ≥ 30 kg/m^2^) has an impact on health. But it is body fat and specifically intra-abdominal fat mass that is the primary determinant of obesity-related morbidity (Dalton et al., 2003). Considering the complexities of the role of diet quality, the preoccupation with caloric restriction and weight loss is not justified by the evidence. It is not uncommon for dietary intervention trials to improve diet quality with improvements in health outcomes, independent of weight change (Itsiopoulos et al., 2011). The predominant focus on obesity as the *primary* dietary related issue could conceivably hinder adoption of the best policy response diverting the focus from diet quality.

There is an accumulating evidence base as to what constitutes a healthy diet. Specifically the health benefits of the Mediterranean diet are now widely confirmed by systematic reviews of intervention studies and cohort studies (Serra-Majem et al., 2006; Sofi et al., 2010). This diet is high in vegetables, legumes, fruits and nuts, fish, unrefined cereals, olive oil, low-to-moderate intake of dairy products and low in meat and “extras.”

Average diet quality is poor in most advanced market economies with their abundant access to highly processed foods, that are strongly marketed. A recent food and nutrition survey in Australia found that only 5.5% of Australian adults have an adequate usual daily intake of fruit and vegetables (Australian Bureau of Statistics, 2014). Over one-third (35.4%) of total energy consumed was from “discretionary foods” of little nutritional value and high in sugars, saturated fats, salt and/or alcohol (Australian Bureau of Statistics, 2013). Diet related diseases continue to rise as reported in the Global Burden of Disease (Lim et al., 2012).

## Aims

Given the poor quality of the average diet, it is clear that current approaches to improving public nutrition are failing. Effective strategies are urgently needed. The remainder of this paper is concerned to understand the reasons for this failure and define the components of an efficient nutrition strategy. As with any behavior change strategy this will need to contain multiple reinforcing elements.

### Policy Elements to Improve Diet Quality

#### Public/Population Health Approaches

***Population level education and information***

The abundance of dietary information freely available from TV advertising, magazine and newspaper articles, blogs, celebrity books and the like, creates confusion among the public about what constitutes a healthy diet. In these forums having a dietary qualification is not a prerequisite for providing dietary advice. The fact that the population, including some health professionals, is unclear about what constitutes a healthy diet is not surprising. The subject is complex and even the scientific literature can be contradictory. Perhaps glaring in this regard has been confusion by the population and health professionals over the “low fat” message, which has had perverse consequences with fats being replaced by sugars (for example in yogurts), often a worse alternative. The complexity is in stark contrast to another behavioral risk factor, that of tobacco smoking, for which the message could not be simpler: “do not smoke.” At the same time the population is exposed to a range of mediums advertising unhealthy foods: TV, print, radio, billboards and point of sale. Such exposure cannot be avoided and is often targeted at children, increasing consumption of low nutrient calorie dense foods. If it did not work, companies would not be spending the billions of dollars on such advertising. Perversely, societies are actually *subsidizing* companies to encourage people to purchase unhealthy snacks and other high sugar foods (including some breakfast cereals, sugary drinks, etc.) through tax deductibility of advertising expenditure (as a business cost).

A number of activities can be employed to improve public information about diet. These include:

• *Social marketing campaigns*, which must be well-designed and draw on evidenced-based information. A core task is to describe the components of a healthy diet—what to eat more of; and what are unhealthy foods to eat less of. In Australia, public nutrition campaigns have been very poorly funded, in effect limiting any possible chance of success and have focused almost exclusively on promoting fruit and vegetable consumption, largely ignoring the issue of unhealthy foods. While successful marketing campaigns (for example to reduce road traffic accidents), have been very well resourced (millions of dollars compared with a few hundred thousand). These campaigns have also been supported by powerful legislative elements—in the case of road traffic accidents including mandatory seat belt legislation, large speeding fines with potential loss of license, all well-enforced.
• *Product labeling* is the second element in public education concerning diet. Developing a system that is informative and correct but also simple has proved elusive, despite considerable research and debate on this issue The core challenge is to create a system of labeling and nutrition information panel that consumers can understand and interpret in the context of their whole dietary pattern. While the simplified systems may seem appealing they all suffer from the complex nature of diet quality.
• *Restrictions on advertising of unhealthy foods*, especially on television and in “children viewing times” has been widely discussed, but rarely adopted. This is despite good evidence that higher TV viewing time is associated with poor diet quality and obesity (National Preventative Health Taskforce, 2009). The policy is viewed as difficult to implement—in terms of defining unhealthy foods and drinks to be targeted and arguments that it will not be effective, given children view television outside of “children viewing times.” There are strong vested interests keen for such a policy not to be adopted.
• *Actively promote dieticians as the best source of expert nutrition advice*. Most individuals no longer know where to find evidence-based nutrition information or which health professionals to trust. Ways of ensuring the integrity of dietary advice provided to the public need to be explored, for example, to confirm the level of evidence supporting specific claims or advice. The public need to be provided with better guidance on where to obtain high-level nutrition information and which health professionals can provide this. (When is your favorite magazine a reliable source of nutrition information?)

***Pricing strategies to promote purchases of healthy food and discourage purchases of unhealthy foods***

Consumers respond to absolute and relative prices; purchases increase when prices fall and reduce when prices increase. Consumers also respond to the prices of complementary or substitute goods—known as the cross-elasticity of demand. For example, if the tax on beer goes up, consumers may reduce consumption but will also switch to wine or spirits. Punitive taxes on unhealthy products could be used to discourage purchases, and subsidies on healthy products could be used to encourage consumption (Black et al., 2013). Food vouchers/stamps are a variant of the latter (Guthrie et al., 2007).

Punitive taxes have been used widely to discourage purchases of tobacco products (and are an efficient, albeit inequitable way of raising revenue). Internationally, most countries apply excise duty on tobacco products. Excise is typically specified as an amount payable per *x* cigarette sticks (Chaloupka et al., 2010). Across the OECD countries, tobacco taxes account for between 43 and 80% of the purchase price of a packet of cigarettes. In 12 of 16 countries tobacco taxes account for over 70% of the purchase price (World Health Organization, 2013, Table 9.2.0 Appendix IX). This equates to a punitive tax of between 230 and 400% on the base price, making cigarettes three to five times as expensive as they would be otherwise. This is a serious policy platform.

Conversations about penalty taxes to change eating behaviors, for example on high saturated fat or high sugar foods have by contrast been canvassing only *very* small tax penalties of around 10–20%. Placing punitive taxes on foods is more problematic than for tobacco products. The logistics are challenging. It is necessary to select the products or food components on which to place punitive taxes, the best method of taxation needs to be determined, e.g., per gram of sugar or fat, or per item falling within designated descriptors. The possible switching of purchases by consumers needs also to be considered. For example, if punitive taxes were placed on drinks with high added sugar, this would increase demand for substitutes such as artificially sweetened drinks, or fruit juices, not necessarily a desired response. The highly contentious tax on saturated fat introduced in Denmark in 2011 and rescinded just 12 months later illustrates the challenge. The fat tax was faced with both political and operational challenges (The Economist, 2012). While some evidence of favorable behavior change was reported (Smed, 2012; Jensen and Smed, 2013), undesirable consequences were also observed, for example with people shopping outside Denmark. A plan to introduce a sugar tax in Denmark was shelved. This is disappointing especially as a tax on sugar makes more sense, in terms of likely health impact than a tax on fat. If implemented it would have created a valuable natural experiment to inform diet policy across the OECD. But again the policy came up against powerful vested interests.

Subsidizing fruit and vegetables and other healthy foods, while common, can have only a small impact on purchases (Bihan et al., 2011; An, 2013). Studies suggest the elasticity of demand for fruit and vegetables is around 0.5 (Powell et al., 2013). Thus, a fall in price of x% would be associated with an increase in consumption of 0.5 ×. This means for example in Australia, where average consumption of vegetables is ∼2 serves per person per day (Australian Bureau of Statistics, 2013), a 30% reduction in price is predicted to increase consumption by 15% (i.e., by 0.3 serves per day). Hence, mean vegetable consumption would increase to 2.3 serves per day, which is still considerably less than the recommended five serves per person per day. That is, the potential size of impact from even quite generous subsidies will be small, but the cost would be considerable in providing the subsidy and in the costs of implementation. Subsidies are logistically challenging to implement. This is not to say that the issue of access to high quality, affordable healthy foods is not important and especially in more isolated communities; but a subsidy on healthy foods is unlikely to be the most effective or efficient way to ensure better access.

Punitive taxes on unhealthy foods could, in contrast have a considerable effect. Demand is more elastic—a price increase on sugar sweetened beverages is predicted to result in at least an equal percent reduction in demand. Extras form a large part of unhealthy diets, so that the effect of a price increase could be substantial. Consider a punitive tax of 50% on soft-drinks, confectionary (including ice-cream), cakes, pastries etc. (that make up ∼80% of extras); this would be predicted to reduce demand for these products by ∼50%. In Australia, where extras constitute 30.6% of the diet, in terms of energy excluding alcohol (Australian Bureau of Statistics, 2013), a 50% fall in consumption would bring extras down to <16%, which is a substantial improvement in diet quality. The policy would also raise revenue. The key challenges are political, given the vested interest that would be affected, and logistical.

***Changing the food environment***

There are a number of possible strategies for changing the food environment as described below:

• *Regulations*—can be promulgated to modify the food environment. Options include (a) proscribing the constituents of food products (e.g., to restrict allowable levels of salt or sugars); (b) the use of zoning provisions, say to limit access to fast food outlets within a defined distance of a school, or to control the density of fast food outlets within vulnerable communities; (c) regulating sale of selected “unhealthy foods” by venue and/or customer (e.g., proscribing foods allowed to be sold or served in school canteens, hospitals, residential facilities or restricting sales of alcohol on premises and take-away by age of customer, hours of opening or customer alcohol history).
• *Working collaboratively with suppliers/industry*—is another option for achieving change in the food supply which may be more politically acceptable and is illustrated by campaigns to reduce the salt content of processed foods. A collaborative salt reduction program in the UK achieved a significant reduction in mean salt intake between 2000/01 and 2011 from 9.5 to 8.1 g per person per day (Department of Health UK, 2012). Agreements to include nutrient supplements in foods have also proceeded through a mix of mandatory and voluntary schemes. Debate continues around the best strategies to fortify breakfast cereals/breads with folic acid (Dalziel et al., 2010).
• *Community-level initiatives*. A wide variety of approaches have been used by communities in an attempt to change the food environment and at the same time actively engage communities in strategies to improve diet quality. Examples include various kitchen garden programs, which demonstrate some success (Hume et al., 2014), programs to make clean fresh cool water more available in schools or in remote communities, and community based obesity prevention programs, such as the French EPODE program (Borys et al., 2012). These programs have mixed success (Dalziel and Segal, 2007).

***Clinical strategies***

Population health strategies can never provide the complete approach to improving the diet. Even with tobacco smoking, which is perhaps the best possible case for population health approaches given the simplicity and clarity of the message, strong public support for legislative intervention and punitive pricing, a substantial clinical program was necessary to achieve the observed behavior changes. These have included funded quit-smoking phone lines, funded quit products (prescription drugs and nicotine patches/products) together with a clear and consistent message from health professionals.

Diet is complex. Simple public health messages can never convey a full understanding of how to construct a healthy diet. Knowledge dissemination in isolation will not achieve sustained dietary improvements. Adopting a better diet requires an understanding of how a current diet might be improved which requires detailed knowledge of the nutritional composition and role of individual foods and food groups, but also contextualized for the individual. Achieving sustained change requires an understanding of barriers and ambivalence to change, but also a knowledge of individual preferences, cultural/cooking traditions, cooking skills, access to foods, food preparation and storage facilities, lifestyle, and family circumstances. This complexity points to the need for individualized dietary advice by a trained and skilled practitioner. A recent review of dietary interventions found that the likelihood of achieving desired behavior change and improved diet quality is greater where qualified dieticians are used (Opie et al., 2013). Accredited Practicing Dieticians are equipped to provide current, evidence-informed nutrition advice and are trained in counseling skills that can address ambivalence and barriers to change, using empowering approaches.

While it is desirable to provide high quality information to the public about healthy diets and a better informed consumer will improve the signals to the food industry (Segal, 1998a,b, Watts and Segal, 2009); population health approaches are not an alternative to funded access to individualized dietary advice and support, but rather a complement.

Most health systems, even those with a strong commitment to universal coverage and public funding, limit subsidized access to dietetic services, whether in the community or hospital in-patient or out-patient settings. Unlike medical and nursing, it is difficult to find international comparisons of access to dietetic services. In Australia, based on the 5-yearly population census that records information on qualification and occupation, in 2011 there were just 2,969 practicing dieticians/nutritionists. This was equal to 1.3 per 10,000 persons, far less than the 5.8 physiotherapists per 10,000 persons or 14.8 General Practitioners (family physicians) per 10,000 persons or 75.0 nurses per 10,000 persons. Most (95%) dieticians/nutritionists are employed in the health/human services sector, many are part-time. Australia thus has approximately one full time equivalent FTE dietician/nutritionist per 10,000 persons. Given the high rates of nutrition related chronic disease and risk which including high blood pressure, high cholesterol, obesity which affect over 50% of adults, it is inconceivable that the current level of dietetic services is adequate.

Additional dietetic positions are needed to ensure access to dietetic services for those with, or at risk of, diet related health conditions. Highly subsidized dietetic services are needed in the community as part of multi-disciplinary primary care teams delivering chronic disease management and prevention and in the hospital in-patient and out-patient settings. If dietetic services are available only on a fee-for-service basis, many in need will fail to access these services, to the detriment of their health, with flow-on implications for the health system and the wider economy. Failure to access multi-disciplinary best practice care is widely reported and funding and delivery arrangements are implicated (Watts and Segal, 2009).

Dieticians need to be available in hospital in-patient and out-patient settings to support patients to adopt healthy eating behaviors. And yet, they are typically regarded as expendable in the face of the inevitable budget pressures. Best practice care would dictate that all cardiac rehabilitation patients, pregnant women (especially those who are obese), mental health patients, persons with diabetes and patients with other conditions for which diet is an established risk factor, have access to individualized professional dietetic services. This simply is not happening in either public or private hospital settings, despite evidence that many dietetic interventions are effective and cost-effective (Dalziel et al., 2006; Dalziel and Segal, 2007).

Dieticians are also needed to work in institutional settings that serve food, many of which involve highly vulnerable populations to improve food quality, for example aged care facilities, preschools, prisons, etc.

Access to high quality professional dietetic advice and support must be a core part of any comprehensive strategy to improve population diet. There is a widespread view that “dietary interventions do not work,” and yet the accumulating evidence of highly successful RCTs suggests the opposite. Success is especially apparent where outcomes are expressed in terms of change in clinical/health outcomes (such as stroke, heart attack, death, incidence of diabetes), rather than intermediate risk markers. Studies with longer term follow-up targeting persons with current diet related disease/risk factors demonstrate particular success, for example in persons with impaired glucose tolerance that report significantly lower rates of progression to type 2 diabetes in intervention group patients (Eriksson and Lindgärde, 1991).

## Major Risks to Implementation

Any attempt to introduce punitive taxes will create winners and losers. The losers will be highly vocal in resisting change, regardless of the potential public benefit. This is the nature of vested interest. Where there is any doubt about the ability to define and target unhealthy foods, political hurdles are magnified. This is illustrated in the Denmark fat tax example. A common response to regulatory approaches is the cry of “nanny state”: that people should be free to make their own choices without interference from government. This response ignores the context in which people make choices. The unfettered behaviors of suppliers and consumers will only be efficient under conditions of the “perfect market.” This requires fully informed consumers with complete knowledge about the products and services available to them and of their impact on their health and wellbeing; free entry and exit whereby no firm can exercise monopoly power (a condition violated by the pharmaceutical industry or in the delivery of clinical services); an absence of externalities—benefits do not extend beyond the consumer or costs beyond the suppliers. This is demonstrably violated in relation to diet, where the wider community bears in part the costs of poor diet quality in higher health care expenditures and lower workforce productivity, costs which suppliers of unhealthy foods fail to bear. There is every reason for governments to intervene. It is demanded by the pervasiveness of market failure (Segal, 2010).

Unfortunately those who stand to gain from an effective strategy to improve diet quality are inevitably less vocal, partly because losses and potential gains are not viewed equally and partly because the general population, which stands to gain, is more diffuse and less powerful. Furthermore the average citizen does not necessarily understand how current distortions are damaging their health. Those who stand to gain most, are persons suffering from, or at risk of, diet related disease, as well as farmers and retailers growing and selling predominantly whole foods. Dieticians would also benefit from expanded employment opportunities. However, none of these groups constitute powerful lobbies.

If punitive taxes were part of the strategy it could be revenue neutral. But if not; it will be said that “there are no funds.” To implement a well-developed public information campaign or expand funding of dietetic services will represent an additional cost. But, diet-related illnesses are the major cost on health budgets and also on the economy in lost productivity, lost production and welfare dependency from premature mortality and disability (from mental and physical illness). In the latest Global Burden of Disease Study (Lim et al., 2012), three of the four top risk factors in advanced western countries are diet related—high blood pressure, obesity and high blood glucose. The potential payoff from improving the eating behaviors of the population is thus considerable. If policies target persons with current diet related conditions, or at high risk, returns on the investment will accrue almost immediately. For example, a dietary intervention to improve eating behaviors in obese pregnant women would deliver health benefits and budget savings within months, through expected lower rates of gestational diabetes and better mother and child outcomes (Thorton et al., 2009; Opie, 2014).

## Conclusion—Core Components of a Nutrition Strategy

It is time to get serious about developing and implementing national nutrition strategies that are capable of effecting change. Multi-component strategies are needed that incorporate social marketing, regulatory restrictions on advertising of junk food/drinks, punitive taxes on unhealthy foods, suitable food labeling and publicly funded dietician services. Dietetic services need to be viewed as part of core heath service delivery and funded at a level that supports access to individualized dietetic services by persons at risk and with current chronic conditions.

While there is considerable pessimism about the ability to improve diet quality across a population, the absence of any comprehensive approach to date, rather gives room for optimism. It is not that we have tried and failed. It is that we have *not* tried and failed. The evidence is clear—diet affects health and is modifiable. Accumulating evidence from intervention studies with long term follow-up demonstrate that eating behaviors can change. But that individualized clinical advice and support is required from highly skilled and trained professionals. If a comprehensive nutrition strategy were adopted, the promise is a substantial improvement in diet quality, better health and wellbeing and lower health care costs. All that is needed is political will and some upfront investment with likely early payoff.

### Conflict of Interest Statement

The authors declare that the research was conducted in the absence of any commercial or financial relationships that could be construed as a potential conflict of interest.
